# Ag Nanocrystals Intercalated Muscovite Mesocrystal for Large-Scale 3D SERS

**DOI:** 10.1021/acs.nanolett.5c02616

**Published:** 2025-07-21

**Authors:** Chia-Yun Sung, Yu-Hao Tu, Le Thi Quynh, Ching-Min Su, Hung-Yi Wu, Lu-Hsing Chen, Kuo-Ping Chen, Wan-Zhen Hsieh, Ching-Yu Chiang, Wen-Hui Sophia Cheng, Ying-Hao Chu

**Affiliations:** † Department of Materials Science and Engineering, 34914 National Yang Ming Chiao Tung University, Hsinchu City 300093, Taiwan; ‡ College of Semiconductor Research, 34881 National Tsing Hua University, Hsinchu City 300044, Taiwan; § Department of Materials Science and Engineering, 34881 National Tsing Hua University, Hsinchu City 300044, Taiwan; ∥ Academy of Innovative Semiconductor and Sustainable Manufacturing, 34912 National Cheng Kung University, Tainan City 701401, Taiwan; ⊥ Institute of Photonics Technologies, Department of Electrical Engineering, 34881 National Tsing Hua University, Hsinchu City 300044, Taiwan; # 57815 National Synchrotron Radiation Research Center, Hsinchu City 300092, Taiwan; ∇ Department of Materials Science and Engineering, 34912 National Cheng Kung University, Tainan City 701401, Taiwan

**Keywords:** Ag nanocrystals, mesocrystal, intercalation, SERS, van der Waals epitaxy, plasmonic nanostructures

## Abstract

Plasmonic nanocrystals represent one of the most fascinating emerging research fields and hold great promise for a wide range of new applications, including surface-enhanced Raman spectroscopy (SERS) and plasmon-related devices. Here, we present a mesocrystal consisting of 3D Ag nanocrystals (NCs) with the same orientation intercalated in a 2D muscovite crystal via a two-step hydrothermal process for a novel SERS platform. The fabricated Ag NCs/mica mesocrystal possesses high crystallinity, uniform size, and extensive distribution to benefit the SERS-active plasmon area and strong plasmon resonance in the visible spectral range. Furthermore, the SERS application potential was demonstrated through Raman spectra of crystal violet and rhodamine 6G molecules on a Ag NCs/mica mesocrystal with detection limits as low as 10^–6^ and 10^–7^ M. This work presents a 3D platform with large-scale uniform hot spots and cost-effectiveness for SERS applications, laying a solid foundation for further investigations into 3D plasmonic nanostructures.

## Graphical abstract

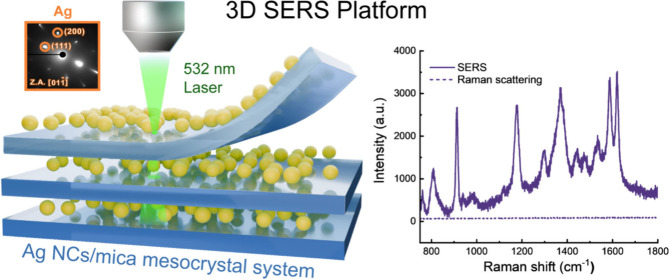

Plasmonic nanostructures have attracted significant scientific interest due to their ability to couple light, generating strong localized electromagnetic fields and inducing localized surface plasmon resonance (LSPR) that can significantly enhance optical processes.^1–6^ The typical feature of a practical plasmonic nanostructure is a rough surface of a noble metal or a high density of metallic nanoparticles.^7,8^ When LSPR is combined with exciton systems, the surface leads to strong surface plasmon exciton coupling, enabling a broad range of applications,^9,10^ including plasmonic biosensing,^11,12^ subwavelength waveguides,^13^ optical antennas,^14,15^ solar harvesting,^16,17^ and surface-enhanced spectroscopy.^18–20^ Among these applications, surface-enhanced Raman spectroscopy (SERS), which offers “fingerprint” information for molecule diagnostics, has gained increasing attention and made significant progress, demonstrating the potential in plasmonic applications across the ultraviolet (UV), visible, and infrared regimes.^16,21–24^ However, despite the progress, developing low-cost, large-scale, and uniform hot spot surfaces for reliable SERS applications remains a challenge, primarily due to the sensitivity of resonance bands to material, dielectric, nanostructure geometry, and crystalline properties.^25^ Thus, providing a high-quality, large-area plasmonic surface with uniformity is crucial for further advancing Raman scattering techniques and broadening their applications.

The most common plasmonic materials are Au, Ag, Al, and Cu. Other metallic materials, such as In, Mg, and Ga, have also emerged as promising candidates for plasmonic applications, offering new directions for research.^26–28^ As for the structures, the extents of different nanostructures have been studied and fabricated over the years. They can be broadly categorized into two main types: solid substrates and suspended metal nanoparticle colloids. Solid substrates are typically manufactured using top-down approaches, such as E-beam lithography or the focused ion beam (FIB) process, allowing for precise nanostructures with well-ordered and designed patterns. However, these processes are expensive, time-consuming, and challenging for achieving large-area coverage. In contrast, the bottom-up methods, such as nanosphere lithography and colloidal Ag nanoparticles, provide a uniform large-scale hot spot area but not an epitaxial relationship with the substrate. Most solid substrate architectures fabricated by the above methods are planar two-dimensional (2D) nanostructures. On the path toward advancing the future of plasmonic materials, there is a clear need for a cost-effective and scalable strategy to construct 3D plasmonic nanostructures for next-generation optical devices and sensing platforms.^29,30^

In this work, we present Ag nanocrystals intercalated mica (Ag NCs/mica) mesocrystals as promising, large-scale, and low-cost platforms for SERS applications. Synthetic muscovite [KMg_3_(AlSi_3_O_10_)­F_2_], referred to as mica, was selected as the 2D host due to its atomic flatness, high thermal and optical stability, and structural flexibility. Its van der Waals gaps act as nanoscale cavities (∼0.1 GPa)^31^ that facilitate van der Waals epitaxy, guiding the oriented growth of Ag NCs along the out-of-plane direction. This process yields highly crystalline Ag NCs with a uniform size (∼98 nm) and high surface coverage (∼39%). Laue X-ray diffraction confirms the epitaxial relationship between Ag(111) and mica(001). The resulting 3D mesocrystal structure generates dense plasmonic hot spots. This enables spatially uniform Raman enhancement, achieving an enhancement factor up to ∼10^6^ at analyte concentrations as low as 10^–5^ M. These findings demonstrate a new pathway for constructing 3D mesocrystals for practical, high-performance SERS sensing applications.

The Ag NCs intercalated mica samples were synthesized through a two-step hydrothermal process. First, AgCl was formed as the silver precursor, followed by thermally assisted decomposition to produce metallic Ag NCs. Fabrication details are available in the Supporting Information. To characterize the Ag NCs in the mica interlayers, the top layer of the sample was peeled off using conventional tapes. We employed scanning electron microscopy (SEM) to examine the sample microstructure. As shown in Figure 1a, Ag NCs grew uniformly and were evenly distributed across the large-scale mica. The Ag NCs can be seen in the selected area of the top-view enlarged SEM image (100000×) and in the EDS mapping of the Ag distribution (Figure 1b and c) to confirm their existence and reveal the detailed morphology. These images demonstrate a one-to-one correlation, indicating the intercalation of Ag NCs within the mica layers. The morphology and spatial distribution of Ag NCs were analyzed by using SEM image processing. The average size, surface coverage (∼80%), and interparticle spacing (a few to 250 nm) were quantified using ImageJ and Python-based OpenCV analysis, as detailed in the Supporting Information (Figures S1 and S2). These regions can serve as hot spots due to enhanced local electric fields.

**Figure 1:**
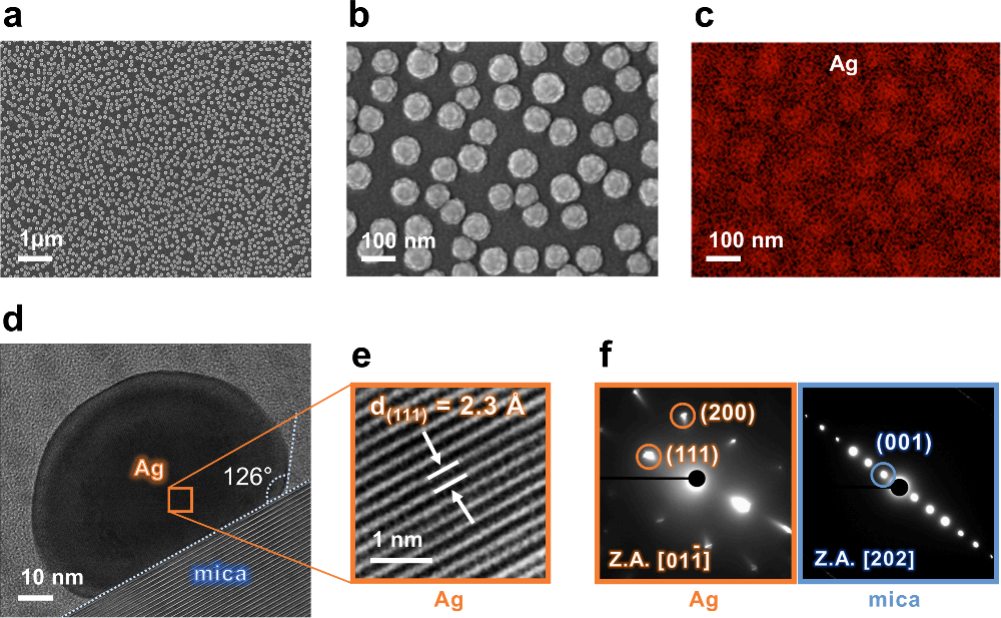
Microstructures of Ag NCs intercalated mica. (a) SEM image of Ag NCs/mica, at a low magnification (15000×). (b and c) Selected area of the top-view SEM image (100000×) of Ag NCs/mica and EDS mapping of the Ag element distribution. (d) High-resolution cross-section TEM image of Ag NCs on the mica crystal. (e) Enlarged image taken from the labeled area of an Ag NC with the *d*-spacing marked. (f) Selected areas electron diffraction of Ag and mica. The diffraction spots imply epitaxy of the Ag NCs.

In the previous work, the mica interlayer was considered a 2D cavity, creating a high-pressure environment.^31^ With this pressure, the growth of the Ag NCs was constrained along the normal axis, which promoted their ordered arrangement, resulting in a 3D-ordered superstructure. The detailed microstructure and interface between an Ag NC and mica were further characterized using high-resolution transmission electron microscopy (HRTEM). The cross-sectional samples for TEM were prepared by FIB. The cross-sectional EDS mapping results (Figure S3) indicate that the Ag NCs were deposited inside the single-crystal 2D mica layers. In Figure 1d, the height of the Ag NC is measured to be ∼38.63 nm. Additionally, this figure provides critical information about the wetting behavior of the two materials. The wetting angle is estimated to be 126°, suggesting low wettability. This finding provides insight into the morphology of the Ag NCs. The flat interface indicates direct contact with the bottom surface. However, the spherical top of the Ag NC suggests a gap between this nanocrystal and the top mica surface. The enlarged image (Figure 1e) taken from the labeled area of the Ag NC shows that the *d* spacing is 2.3 Å. Furthermore, the selected area electron diffraction image (SAED) patterns with the [202]_mica_ zone axis displayed in Figure 1f indicate that the lowest surface energy plane of the Ag NC is the {111} plane. These results suggest that the Ag NC is epitaxially intercalated within the mica layers. Figure S4a provides a schematic illustration of the crystallographic orientation relationship between the two materials.

From the previous section, we discovered that the Ag NC exhibits an epitaxial relationship with mica. Consequently, we propose that all of the Ag NCs grew in the same direction, considering the potential of this material being a mesocrystal, and a new class of nanostructured solids composed of numerous tiny crystals of similar size and shape was arranged in the same orientation. To investigate the crystal orientation of Ag NCs on a large scale, the samples were analyzed using X-ray diffraction (XRD). The X-ray θ–2θ scan results (Figure 2a) indicate that the Ag NCs grew along the [111] direction. The Ag(111) and Ag(222) diffraction peaks appear at approximately 37.86° and 81.37°, respectively, which match with the American Mineralogist Crystal Structure Database (amcsd-0011135). This result illustrates the alignment of Ag nanocrystals without other orientations in the mica crystal. According to Bragg’s law, the measured interplanar distance for Ag is 2.38 Å for *d*_(111)_, consistent with the TEM results. To examine the symmetry and in-plane orientation relationship of the Ag NCs intercalated in mica, ϕ scans of the Ag(220) and mica(202) reflections were conducted, as illustrated in Figure 2b. The peaks in the ϕ scans of Ag(220) reflections show well-defined epitaxial relationships concerning the mica crystal. Six symmetric peaks in the corresponding ϕ scans of Ag(220) reflections indicate that the epitaxial Ag NCs have two sets of orientations in the in-plane direction. The ϕ scan also reveals the in-plane orientation relationships between Ag and the mica crystal as (111) [11̅0]_Ag_//(001)­[010]_mica_, in agreement with the TEM diffraction results. Furthermore, crystallinity is evaluated through the ω scan (rocking curve) at Ag(111), which has a full width at half maximum (fwhm) as narrow as ∼0.23° (as shown in Figure 2c).

**Figure 2:**
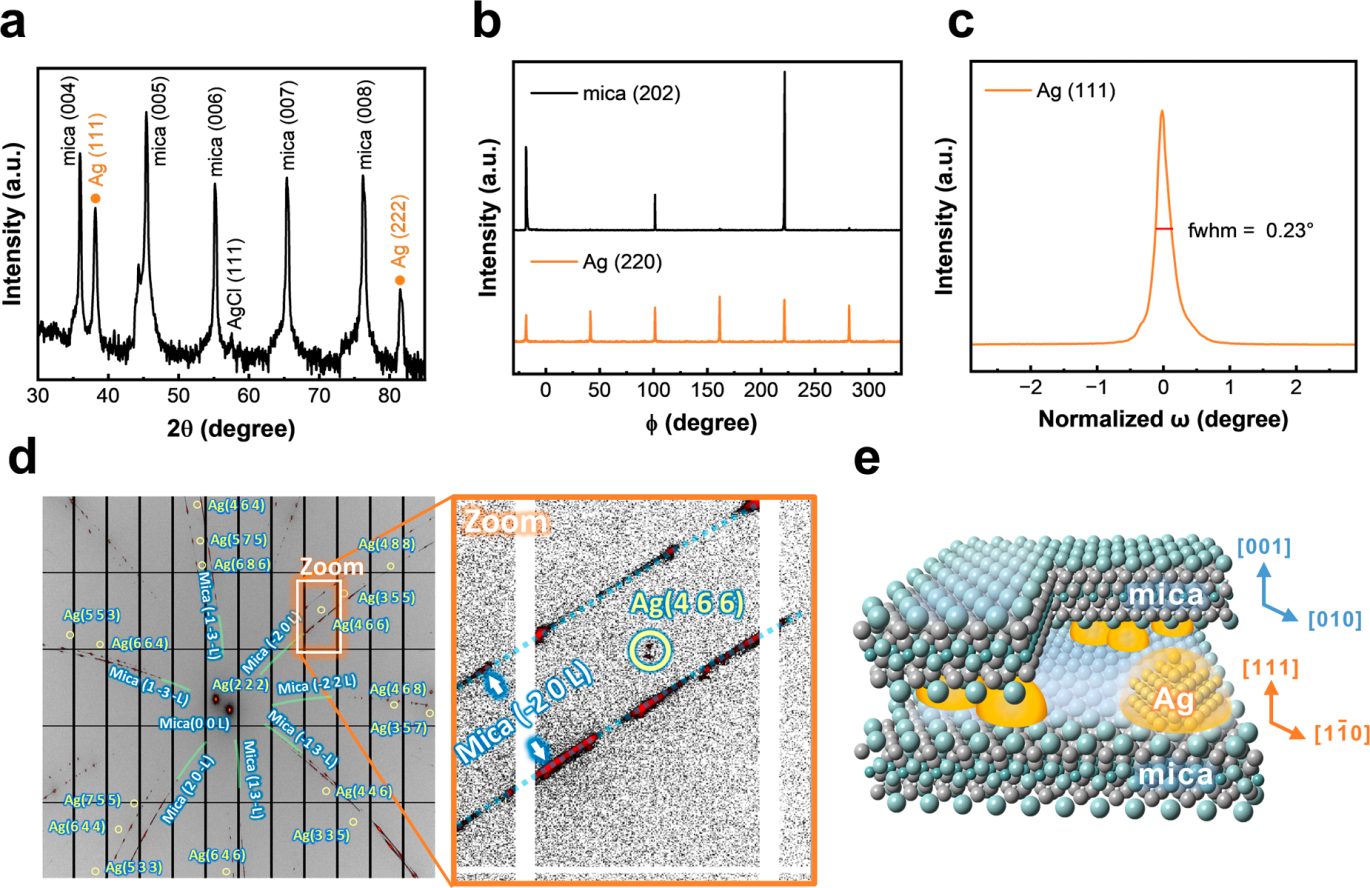
Structural characterization of the epitaxial Ag NCs/mica mesocrystal system. (a) XRD θ–2θ scan result of Ag NCs/mica. (b) In-plane ϕ scan of Ag(220) and mica(202). (c) ω scan for Ag(111), showing a narrow fwhm of 0.23°. (d) Laue diffraction shows the Ag in-plane and out-of-plane relationships with the mica crystal. (e) Schematic growth relationship of 3D Ag nanocrystals grown into 2D mica layers, forming the (111) Ag nanocrystal-intercalated (001) mica layers.

These results prove that all of the Ag NCs are arranged in the same orientation, a critical indicator for mesocrystal formation. However, standard XRD has limitations due to the source power. Therefore, we further conducted synchrotron-based Laue diffraction measurements to verify this hypothesis. The results presented in Figure 2d, along with fitted Miller indices (*hkl*), reveal the single-crystalline feature of the Ag nanocrystals intercalated with mica, delivering evidence of superior crystallinity. Consequently, this unique 3D-ordered superstructure can be described as a Ag NCs/mica mesocrystal (Figure 2e), which can benefit from a significant plasmon LSPR. To evaluate its optical properties, dielectric functions were extracted from spectroscopic measurements (300–1000 nm), as detailed in the Supporting Information (Figure S5). The data reveal that the Ag NCs exhibit low optical loss^12,32,33^ and support enhanced Raman scattering due to large-area hot spot formation.

We expect to observe a near-field electromagnetic enhancement on the 3D Ag NCs/mica mesocrystal, as illustrated schematically in Figure 3a. To investigate this effect, two commonly used organic dyes, crystal violet (CV) and rhodamine 6G (R6G), were selected as target molecules. First, the UV–vis absorbance spectra of Ag NCs combined with CV and R6G were measured, along with the corresponding reference samples containing only CV and R6G. The background signal from the mica was subtracted. As shown in Figure 3b, the absorption peak of the Ag NCs is located at 442 nm, corresponding to the NC size.^34^ Besides, when Ag NCs are combined with molecule dyes, the absorption peak becomes broader, which can be attributed to the coupling between Ag NCs and molecules. One should be noted that the LSPR resonant shift can also be attributed to the change of the environment index around metal NCs. The detailed trends with an increasing molecule concentration of CV and R6G are shown in Figure S6a and b. The absorption should overlap with the laser excitation wavelength to achieve strong SERS signals. In this work, a 532 nm laser with a spot size of ∼350 nm is chosen for the SERS study. The Raman spectra of CV and R6G aqueous solutions have different concentrations. For CV, concentrations from 10^–5^ to 10^–8^ M were used, as shown in Figure 3c. For R6G, the range was from 1 × 10^–4^ to 1 × 10^–7^ M, as shown in Figure S6c. The SERS spectra of both molecules display clear and identifiable characteristic peaks, with the signal intensity gradually decreasing as the concentration of CV molecules decreases. The peak positions correspond well to their Raman spectrum, confirming molecular identification. The detection limit of CV and R6G molecules was detected at 10^–7^ and 10^–6^ M.

**Figure 3:**
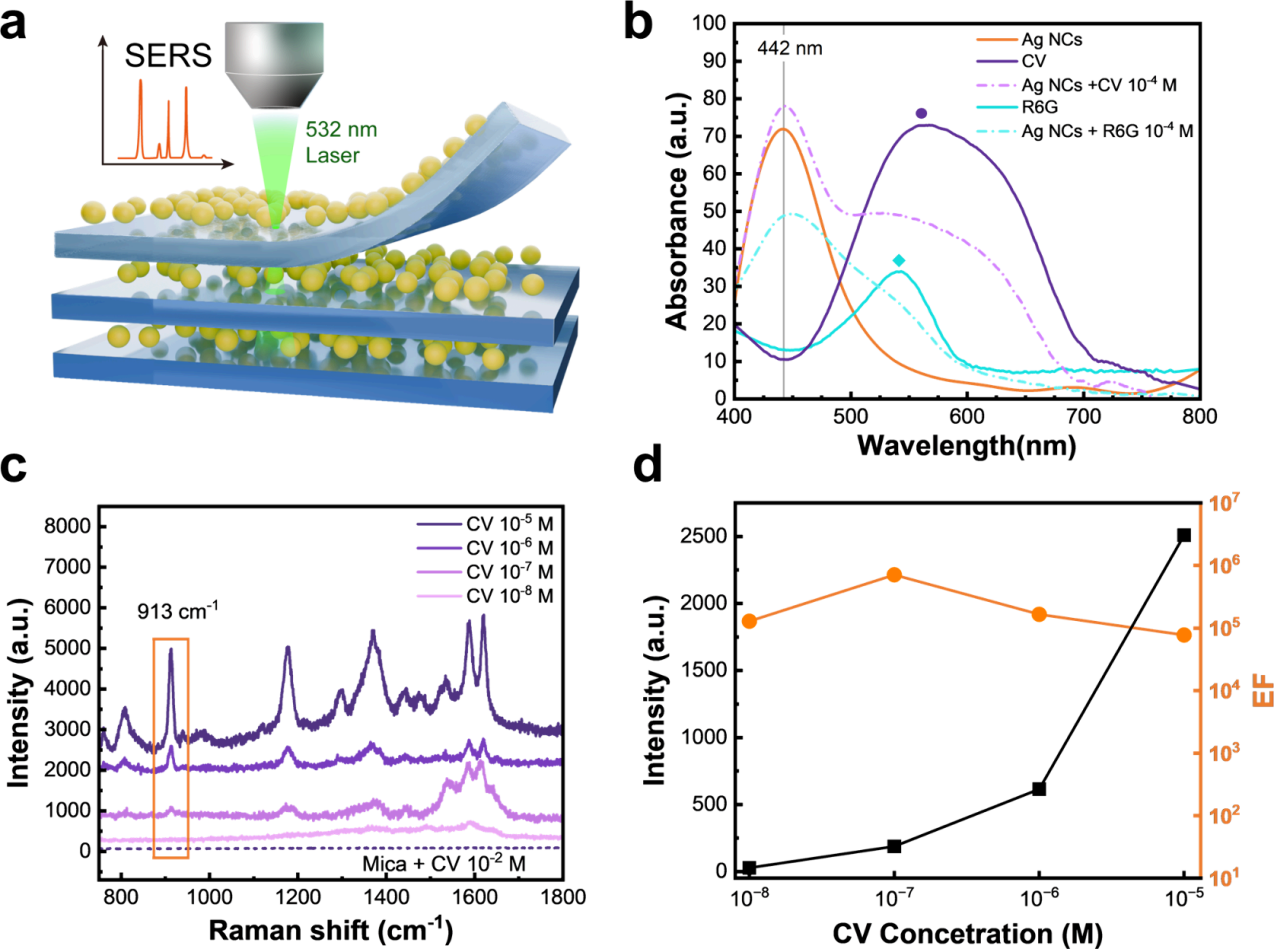
SERS experimental results of Ag NCs intercalated with mica. (a) Schematic representation of the working mechanism of the SERS platform. (b) UV–vis absorbance spectra of Ag NCs, Ag NCs combined with two different dyes, and each dye alone. The background signal from the mica has already been subtracted. It indicated that the LSPR resonance of Ag NCs is 442 nm. (c) SERS performance of CV/Ag NCs/mica. The Raman signal was obtained from Ag NCs/mica and pure mica crystal. The excitation laser is a 532 nm solid-state laser; the integration time is 20 s. The 532 nm excitation laser has a spot size of 350 nm. (d) Change of the SERS intensity for various concentrations of CV molecules at 913 cm^–1^ peak (black curve) and calculated enhancement factor (EF) of CV on the Ag NCs/mica SERS system (orange curve).

To quantify the enhancement effect, the SERS intensity and corresponding Raman enhancement factor (EF)^18^ versus the concentration of CV and R6G SERS spectra are plotted in Figure 3d and Figure S6d. The same concentration ranges were used for the EF evaluation. A reference concentration on bare mica (*C*_RRS_) was set at 10^–2^ M for both CV and R6G, indicated by dashed lines in Figure 3c and Figure S6c, respectively. The Raman EF is ∼10^6^ for 10^–5^ M CV at 913 cm^–1^ and ∼10^5^ for 10^–6^ M R6G at 613 cm^–1^. Table S1 compares the Ag NCs/mica system to other Ag nanostructures under green laser excitation, showing that the Ag NCs/mica system can achieve a high enhancement factor.

To further evaluate the uniformity and reproducibility of the SERS performance of the Ag NCs/mica mesocrystal, SERS spectra were acquired from three random positions on each of three different batches of Ag NCs/mica, resulting in a total of nine spectral measurements. The shape of the SERS spectra and their peak intensities exhibit high similarity, indicating the consistency of the samples and demonstrating the stability of the SERS performance (Figure 4a and b). In addition, the relative standard deviation (RSD) of signal intensity was obtained to be 10.6% at 913 cm^–1^.

**Figure 4:**
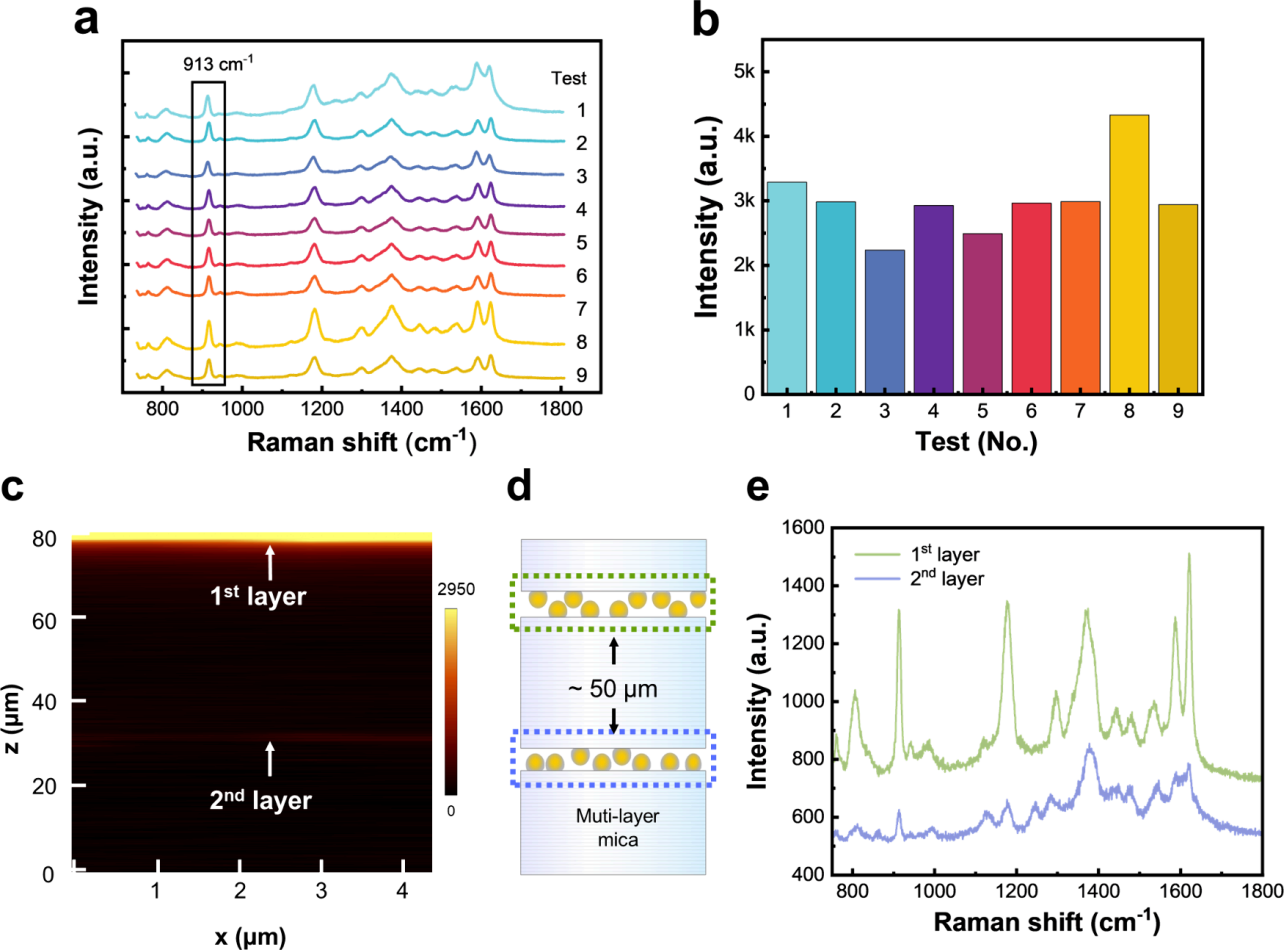
Corresponding SERS mapping performance. (a) SERS spectra were collected from three randomly selected points on each of the three CV/Ag NCs/mica samples. (b) Histogram of the 913 cm^–1^ peak intensities plotted from the SERS spectra. (c) Raman *x*–*z* direction mapping of CV molecules on the Ag NCs/mica. The scan covered a 5 μm width along the *x*-axis and a 100 μm depth along the *z*axis. (d) Schematic represents the Ag NCs intercalated in the mica crystal layers. (e) SERS spectra of CV/Ag NCs/mica at the first Ag NCs interlayer and the second Ag NCs interlayer.

Using this SERS platform design, large-area 10^–5^ M CV/Ag NCs SERS can be distinguished by Raman intensity mapping because of spatially uniform Ag NCs over a large surface area, preventing the formation of an inhomogeneous surface. The vertical distribution of the SERS Ag NCs/mica is also investigated. The Raman *x*–*z* direction mapping of CV molecules (10^–5^ M) on the Ag NCs/mica mesocrystal is shown in Figure 4c. Raman mapping was conducted using a WITec alpha300R confocal microscope (532 nm), providing sub-300 nm lateral and sub-900 nm axial resolution (details in the Supporting Information). The scan covered a 5 μm width along the *x*-axis and a 100 μm depth along the *z*-axis, with an integration time of 1 s per pixel. This *z*-axis Raman mapping revealed two layers of Ag NCs coupled with CV molecules, with an interlayer distance of approximately 20–50 μm. Figure 4d represents the schematic of the cross-sectional structure of the Ag NCs/mica mesocrystal. Due to the *z*-axis, these results reflect macroscopic features obtained from large-area Raman mapping, demonstrating a large active area and high uniformity of the Ag NCs/mica mesocrystal. However, the distance between each intercalated layer is much larger than the incident wavelength, which weakens the detectable Raman signal, as shown in Figure 4e. Therefore, this factor should be considered when developing the SERS platform. The 3D Ag NCs/mica mesocrystal SERS platform can extend to the SERS-active side. The advantage of the large hot zone and uniformity of the SERS platform stems from the high quality of Ag NCs intercalated in the mica. This leads to reduced intrinsic loss and support of a strong surface plasmon, offering the potential for developing practical 3D sensing applications.

To understand the origin of the plasmonic resonance of the Ag NCs/mica mesocrystal system, 3D finite-difference time-domain (FDTD) simulations were reported. The results are shown in Figure 5. The simulated plane view and cross-sectional view of the electric field with a mica thickness of 700 and 50 nm are visualized. A pronounced field enhancement driven by LSPR at the Ag NCs sites can be observed. The coupling between neighboring nanoparticles leads to a more intense field. Notably, the simulation field profile reveals significant insights into the interaction of dielectric mica layers with intercalated Ag NCs. As the mica layer is reduced in thickness, the field enhancement at the top Ag NCs becomes even more pronounced, reflecting the increased coupling efficiency between Ag NCs in both the lateral and vertical directions. In contrast, when the mica layer exceeds the wavelength of the incident light, the field intensity diminishes significantly, indicating weaker plasmonic interactions and a reduced confinement of the electromagnetic field within the Ag NCs regions. As shown in Figure 5b and c, the observed variations in dipole orientation primarily originate from near-field coupling between neighboring Ag NCs, which alters the local electromagnetic environment. To validate this, control simulations with isolated particles were conducted, confirming that such deviations result from interparticle interactions rather than changes in the material or excitation parameters. Furthermore, the effect of mica thickness on near-field enhancement was evaluated through hotspot density analysis. Detailed simulation procedures and quantitative comparisons are provided in the Supporting Information (Figure S7).

**Figure 5:**
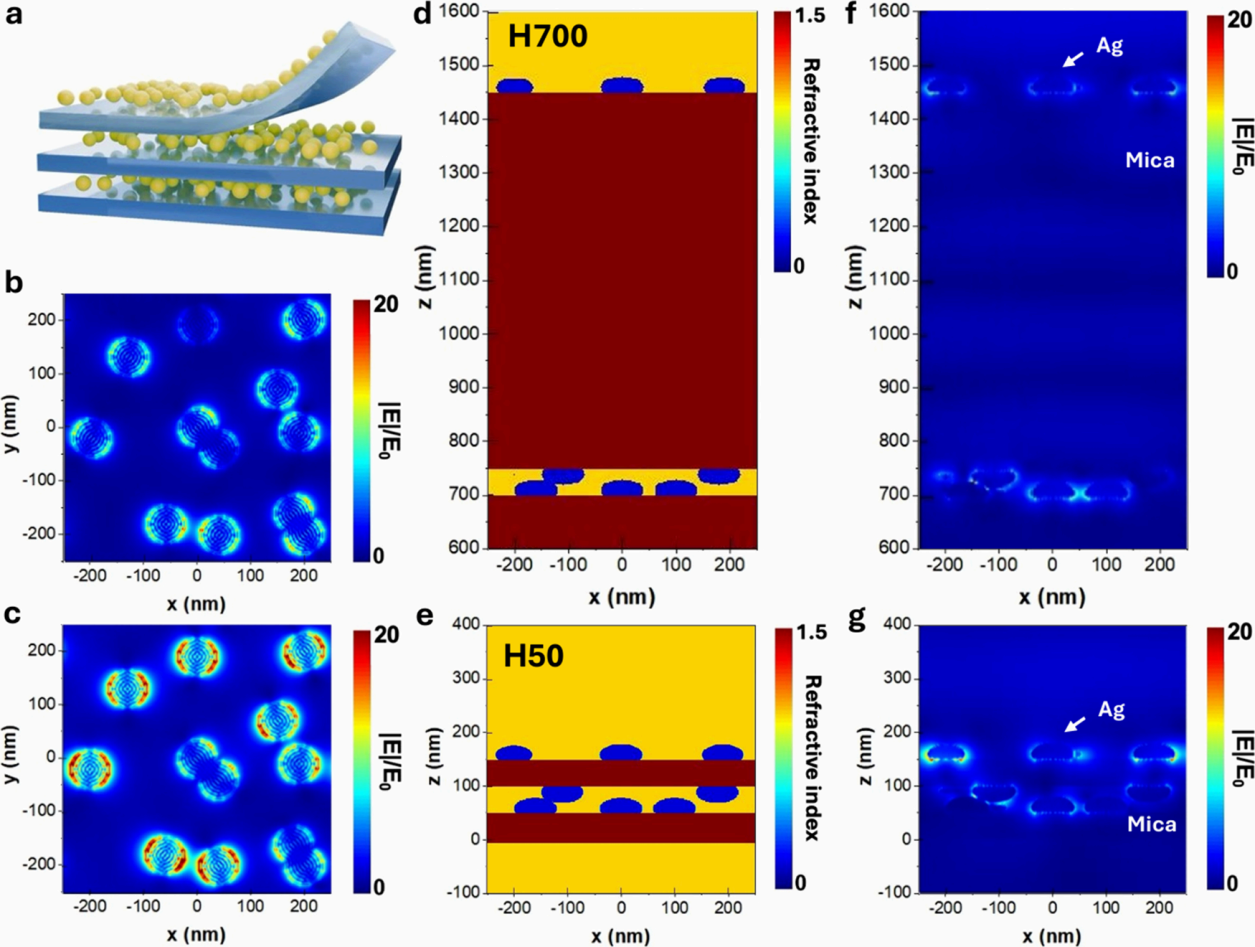
Simulated electric field distribution of the 3D Ag NCs/mica at 532 nm. (a) Schematic diagram of the 2D/3D/2D intercalation structure. (b) Simulated plane view of the electric field at the top of Ag NCs with a mica thickness of 700 nm. (c) Simulated plane view of the electric field at the top of Ag NCs with a mica thickness of 50 nm. (d) Cross-sectional view of the index monitor with a mica thickness of 700 nm. (e) Cross-sectional view of the index monitor with a mica thickness of 50 nm. (f) Simulated cross-sectional view of the electric field with a mica thickness of 700 nm. (g) Simulated cross-sectional view of the electric field with a mica thickness of 50 nm.

Due to the dense hotspots characterized by high spatial confinement and intensity enhancement, the Ag NCs/mica mesocrystal becomes a desirable platform for SERS, where the Raman scattering cross-section is directly influenced by the fourth power of the local electric field. This underscores the critical role of precise control over the mica thickness in achieving optimal plasmonic coupling and resonance conditions.

The findings highlight the potential of mechanically exfoliated or chemically thinned mica sheets as tunable dielectric spacers in hybrid plasmonic architectures. By minimization of the interlayer spacing, stronger near-field interactions between Ag NCs can be achieved, forming a coupled plasmonic system that amplifies the local field intensities. This simulation demonstrates that a platform based on thin mica layers with embedded plasmonic nanoparticles offers a powerful route toward high-performance SERS substrates. The ability to modulate field strength through proper control of the dielectric spacing paves the way for new advances in molecular sensing, label-free detection, and nanophotonic device engineering.

We developed a synthesis strategy for 3D Ag NCs intercalated in 2D mica to form a mesocrystal structure, offering a promising platform for uniform, stable, and large-area 3D SERS applications. SEM and TEM–SAED revealed highly uniform morphology and confirmed the epitaxial alignment of Ag(111) NCs with mica(001), further supported by Laue diffraction and XRD ϕ-scan analyses. SERS measurements using R6G and CV demonstrated detection limits down to 10^–6^ and 10^–7^ M, with enhancement factors up to 10^6^. This 3D hybrid mesocrystal structure holds a strong potential for scalable fabrication and application in environmental monitoring and biosensing.

## Methods

The experimental details are described in the Supporting Information.

## Supplementary Material



## Notes

Detailed methods of sample fabrication, characterization, and simulation, hydrothermal synthesis process of Ag NCs/mica, including the characterization of their structural and optical properties, Python algorithm used for analyzing Ag NCs spacing, enhancement factor (EF) calculations and spectroscopic ellipsometry (SE) measurements to evaluate optical properties, statistical analyses of particle size and spacing based on SEM images (Figure S1), optical image and corresponding SEM images of the Ag NCs/mica sample, along with a schematic of intercalated Ag NCs (Figure S2), cross-sectional SEM and EDS–SEM images confirming the distribution of Ag NCs and identifying key elements in mica (Figure S3), schematic of the growth orientation of Ag NCs on mica and AFM image of the mesocrystal film (Figure S4), dielectric function of Ag NCs/mica with reference data (Figure S5), optical absorbance spectra, SERS results for different dye concentrations, and corresponding EF values (Figure S6), and calculated enhancement factors (Table S1) (PDF)
