# Supplementary material for: Ag Nanocrystals Intercalated Muscovite Mesocrystal for Large-Scale 3D SERS
Source: Nano Lett. 2025 Jul 21;25(30):11632–9. doi: 10.1021/acs.nanolett.5c02616 (PMC12314913; doi:10.1021/acs.nanolett.5c02616)
Supplement: Supplementary file 1 [file nl5c02616_si_001.pdf]

# Ag Nanocrystals Intercalated Muscovite Mesocrystal for Large-Scale 3D SERS

Chia-Yun Sung[1], Yu-Hao Tu[2], Le Thi Quynh[3], Ching-Min Su[1], Hung-Yi Wu[4], Lu-Hsing Chen[5], Kuo-Ping Chen[5], Wan-Zhen Hsieh[6], Ching-Yu Chiang[6], Wen-Hui (Sophia) Cheng[4,7*], and Ying-Hao Chu[1,2,3*]

[1]Department of Materials Science and Engineering, National Yang Ming Chiao Tung University, Hsinchu City 300093, Taiwan

[2]College of Semiconductor Research, National Tsing Hua University, Hsinchu City 300044, Taiwan

[3]Department of Materials Science and Engineering, National Tsing Hua University, Hsinchu City 300044, Taiwan

[4]Academy of Innovative Semiconductor and Sustainable Manufacturing, National Cheng Kung University, Tainan City 701401, Taiwan

[5]Institute of Photonics Technologies, Department of Electrical Engineering, National Tsing Hua University, Hsinchu City 300044, Taiwan

[6]National Synchrotron Radiation Research Center, Hsinchu City 300092, Taiwan

[7]Department of Materials Science and Engineering, National Cheng Kung University, Tainan City 701401, Taiwan

*Correspondence to: yhchu@mx.nthu.edu.tw and wcheng@gs.ncku.edu.tw

# Method

## Hydrothermal Synthesis of Ag nanocrystals intercalated mica

Ag NCs intercalated mica were prepared using a two-step hydrothermal growth method. First, a 2M NaCl solution was prepared by dissolving NaCl (99.9%, purchased from Seedchem) in deionized water. The solution and a certain amount of pre-cut 5×5 mm mica crystals were transferred into a Teflon-lined sealed stainless-steel autoclave and maintained at 100℃ for 24 hours. During this step, the sodium and chloride ions enter the mica interlayers with water molecules, creating a thin aqueous solution layer. When the autoclave naturally cools to room temperature, the crystals will be removed, and the excess surface residue will be wiped off. After that, they were immediately immersed in a 0.1 M $AgNO_3$(99.8%, Aencore Chemical) aqueous solution and heated in an autoclave again at 60°C for 24 hours. A precipitation reaction occurs when the silver ions in the $AgNO_3$ solution encounter the residual NaCl solution between the layers.

$$Ag^{+} + Cl^{-} \rightarrow AgCl$$

AgCl NCs precipitated within the layered structure of mica, forming AgCl-intercalated mica crystals (AgCl/mica). Finally, the AgCl/mica is heated to 500°C at a ramp rate of 10°C per minute in a reducing atmosphere and held at this temperature for 6 hours. During this process, AgCl decomposes into metallic silver and chlorine gas.

$$2AgCl \rightarrow 2Ag + Cl_2$$

Silver nucleates and grows within the mica interlayers, resulting in uniformly sized nanocrystals that create the Ag nanocrystals intercalated mica (Ag NCs/mica). The chlorine gas may carry Ag vapor across the mica surface, resulting in a more uniform spatial distribution. Since this study focused on the Ag NCs grown in the mica interlayers, the top and bottom layers of the Ag NCs/mica were removed by commercial paper label stickers, revealing the Ag NCs inside. This approach offers the unique features of low cost, high quality, and high uniformity over a large surface area of nanostructures.

**Preparation of aqueous solution for SERS on Ag/mica multilayer surface**

The Rhodamine 6G (R6G) was purchased from Thermo Fisher Scientific Inc., while crystal violet (CV) was acquired from Macklin Inc. To assess the SERS performance of Ag NCs/mica mesocrystal, R6G and CV were each dissolved in DI water at concentrations ranging from $10^{-4}$ to $10^{-7}$ M and $10^{-5}$ to $10^{-8}$

M, respectively. The solutions were prepared using an ultrasonic bath to ensure the complete dissolution of the solutions. The Ag NCs/mica mesocrystals were soaked in the solutions for 15 minutes before the UV and Raman spectra were measured.

**The Structure Characterization**

The surface morphology of the samples was examined by a Schottky field emission scanning electron microscope, JSM-IT800 (SEM). A high-resolution X-ray diffractometer (XRD, Bruker D8 ADVANCE) was used to assess crystal quality and epitaxial relationships. The specimens for transmission electron microscopy (TEM) in the cross-sectional configuration were prepared using a focused ion beam (FIB, FEI Helios Nanolab 600i). The Ag NCs/mica mesocrystal crystal structure was characterized using a spherical-aberration corrected field emission transmission electron microscope (ULTRA-HRTEM).

**Ag NCs spacing analysis algorithm**

To accurately analyze the distance distribution of Ag NCs, an image processing algorithm was developed using OpenCV in Python. The goal was to identify individual nanocrystals and measure the distances between their edges. The algorithm includes the following steps:

1. Image Preprocessing:
   The raw SEM image was converted to grayscale and binarized using Otsu's thresholding method to enhance contrast and isolate NCs from the background.

2. Edge detection:
   The edges of the NCs were detected using the Canny edge detection algorithm, which provides boundary identification.

3. Contour identification:

   To calculate the dimensions and positions of each NC, all NCs were defined as ellipses, and the contours were extracted by the 'findContours' function in OpenCV.

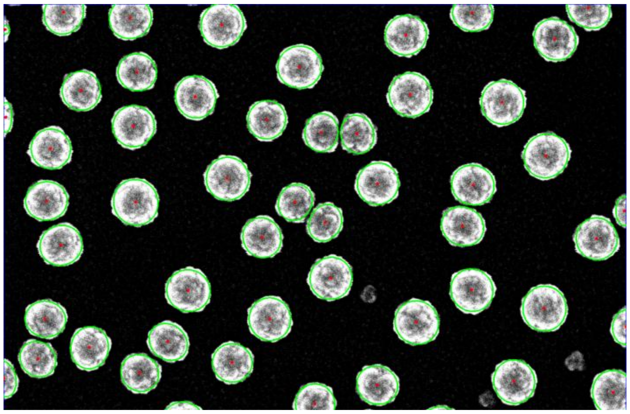

4. Neighbor Definition via Voronoi Diagram:

   A Voronoi diagram was generated based on the centroids of the detected nanocrystals to define neighboring nanocrystals. The diagram partitions the space into regions, where each region corresponds to the area closer to a specific nanocrystal than to any other. Neighboring nanocrystals were defined as those sharing common boundaries in the Voronoi diagram. This method provides an accurate and visualization-friendly way to identify interparticle relationships.

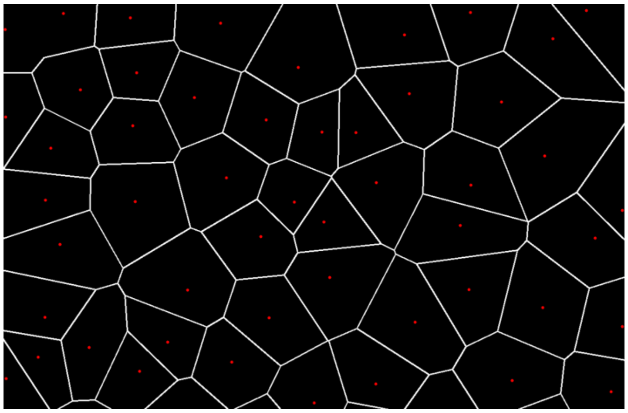

5. Distance Calculation:

   The center-to-center and edge-to-edge distances between nanocrystals were computed using Euclidean distance formulas. Distances between neighboring

nanocrystals, as defined by the Voronoi diagram, were prioritized in the analysis. The results revealed hot spots where interparticle spacings were less than 10 nm.

6. Data Output:

The calculated spacing data were compiled into histograms and overlaid on the original SEM images to visually validate the results. Figures S1c and S1d show examples of the processed images, Voronoi diagram visualizations, and spacing distributions.

**Nano X-ray Tools in the Synchrotron**

We conducted measurements using X-ray nanobeam diffraction spectroscopy at beamline 21A, Taiwan Photon Source (TPS), at the National Synchrotron Radiation Research Center (NSRRC), Taiwan. The core technique of this beamline employs a white-beam X-ray with an energy range of 5 to 30 keV to capture the Laue diffraction pattern for determining crystal structure, orientation, and strain/stress. The Laue diffraction images were produced by accumulating diffraction peaks from the sample region. An 80 nm X-ray beam was scanned over a 3 μm range in the x-direction, with the Laue diffraction signal recorded for 3 seconds at each position. The spotty diffraction patterns observed in the ring-shaped formations arise from several Ag nanocrystals within a sampling distance of 3 μm. Consequently, the strong diffraction points along the diffraction lines of Ag indicate that the porously grown Ag nanocrystals exhibit preferential orientations. The beamline utilized a fixed-shape Kirkpatrick-Baez mirror to focus the X-rays to 80 nm and a scanning stage to facilitate 2D mapping.

**Absorbance Measurements**

The absorption spectra of the Ag NCs mesocrystal covering the wavelength range of 500 to 900 nm were measured using a Kymera 328i spectrometer (Andor Technology) equipped with a DU401A-BVF CCD detector, coupled through a 20× microscope objective. The unpolarized broadband light was provided by a halogen tungsten lamp, and measurements were performed in transmission mode.

**SERS measurements**

The samples were immersed in dye solutions for 15 minutes and then dried in an electronic humidity control cabinet before SERS measurement. The Raman signal was acquired using

a confocal Raman Imaging Microscope (WITec alpha300R, Oxford). A 1 mW, 532 nm diode laser excited the sample through a 100× objective lens (N.A. = 0.9, Olympus, MPlanFL) with a laser spot size of approximately 350 nm. All measurements were conducted at room temperature.

**Enhancement Factor (EF)calculation**

The enhancement factor for SERS platform is calculated using the following equation:[1,2]

$$\text{Enhancement Factor} = \frac{\frac{I_{SERS}}{C_{SERS}}}{\frac{I_{RRS}}{C_{RRS}}}$$

, where $C_{SERS}$ and $C_{RRS}$ are the concentration of molecules contributing to the SERS and Resonance Raman Scattering (RRS) signal intensity on bare mica. $I_{SERS}$ and $I_{RRS}$ are the SERS and RRS signal intensities, respectively.

**Spectroscopic Ellipsometry (SE)**

The SE measurements were performed using a SENresearch 4.0 spectroscopic ellipsometer. The focusing probe attachment provides an incident spot size of ∼300 μm. The Ag thin films/mica and Ag NCs/mica were measured at an angle of 75° with respect to the normal plane of the film surface. The collected wavelengths range from 300 nm to 1000 nm. The dielectric function of thin films was obtained by fitting with the Drude-Lorentz model.

**x-z Raman measurements**

The x-z Raman signal mapping was obtained by the stage scan with a lateral resolution of <300 nm and a depth resolution of <900 nm at 532 nm excitation. With the single-mode fiber as the pinhole to collect the Raman spectra, the out-of-focus signal could be eliminated. The absorption coefficient α at λ=532 nm can be calculated to be 1.18 $cm^{-1}$. This corresponds to an attenuation length is around 847 μm, indicating that less than 12% of the incident light is attenuated over 100 μm.

**Simulation**

In this simulation, we utilized Lumerical FDTD software for its accuracy in predicting electromagnetic wave interactions with the 3D Ag NCs/mica structure. We constructed a

cuboid simulation space with a width of 500 nm. Within this space, we placed Ag NCs randomly on the surface of the exposed mica, representing the SEM images obtained from the experiment. The optical indices of Ag NCs and mica were measured by Spectroscopic Ellipsometry (SE). We note that additional meshes with $2\times2\times2$ $nm^3$ were employed to balance computational efficiency and accuracy of shape. To model the larger arrays, we applied periodic boundary conditions in the x and y directions. In the z-direction, we incorporated two perfectly matched layers to absorb incoming waves and prevent reflections. To excite the 3D Ag NCs/mica structure, we introduced a linearly polarized light source propagating in the negative z-direction, allowing us to analyze the optical properties. We positioned monitors at x-y and x-z cross-sections to examine the electric field distributions at 532 nm.

## Supporting Figures

The size distribution shown in **Figure S1a and S1b** was analyzed using ImageJ software, with the mean Feret diameter of the Ag NCs measured at 98 nm. The corresponding relative standard deviation value of particle size was calculated to be 9.3%, and the Ag NC coverage reached 38.9% on one side of the interlayer. Based on **Figure S2**, the distribution of nanoparticles appears to be symmetric. Therefore, the overall coverage can be estimated as twice this value, close to 80%, suggesting a superior interface coverage. As a plasmonic material, the distance between NCs is one of the crucial factors for local electric field enhancement. A Python algorithm based on OpenCV was developed for image processing to calculate the distances between Ag NCs. The visualized result and the distance distribution are shown in **Figure S1c, d**, showing the spacings ranging from a few nm to 250 nm. These regions can serve as hot spots due to enhanced local electric fields.

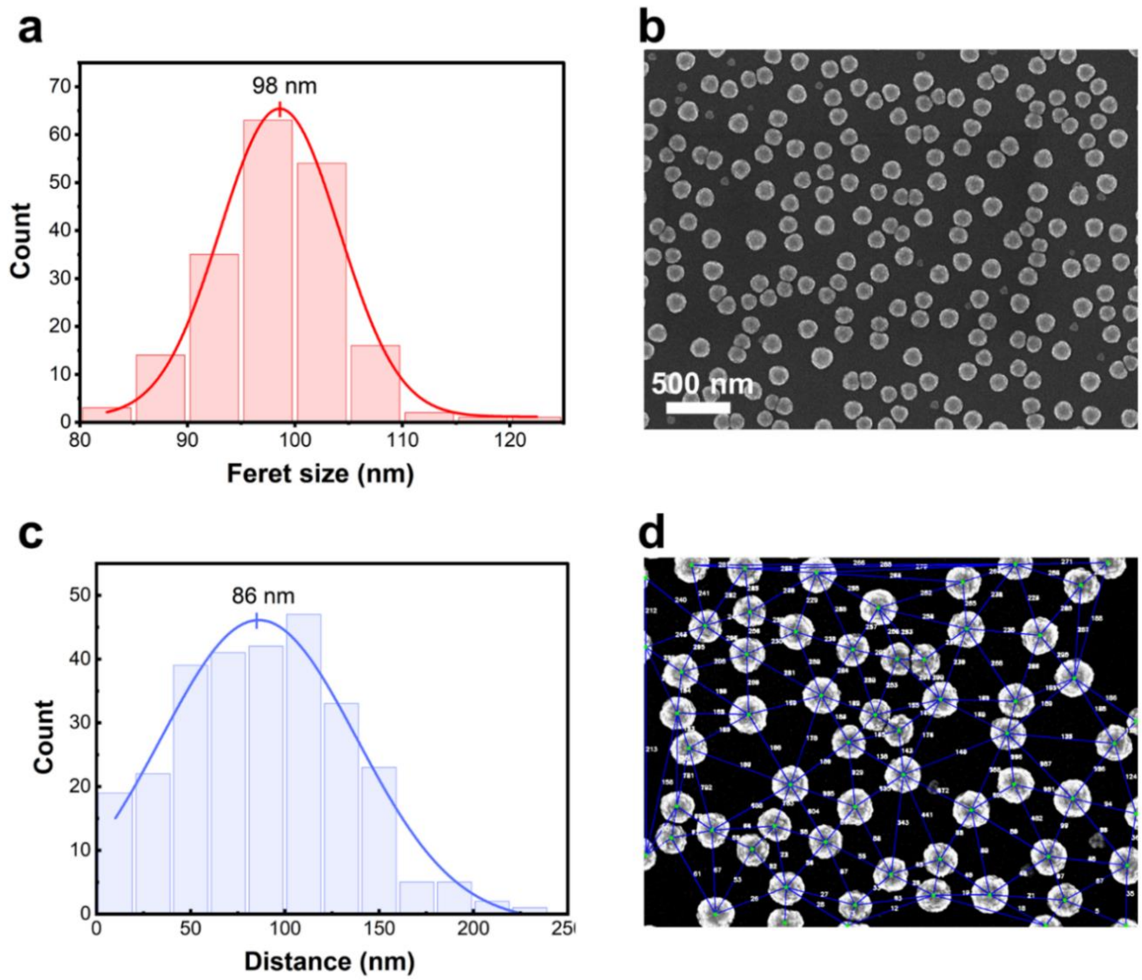


**Figure S1. Statistics on particle spacing and size.** (a) The size distribution of Ag NCs is obtained by software ImageJ from (b) the top-view SEM images of the Ag NCs/mica. The particle size was calculated by software ImageJ from (b) the top-view SEM images of the Ag NCs/mica over a large area (5×5 $\mu m^2$). The corresponding relative standard deviation value was calculated to be 9.3%. (c) The distance distribution of Ag NCs from the SEM image (d). Visualization of the distance statistical results.

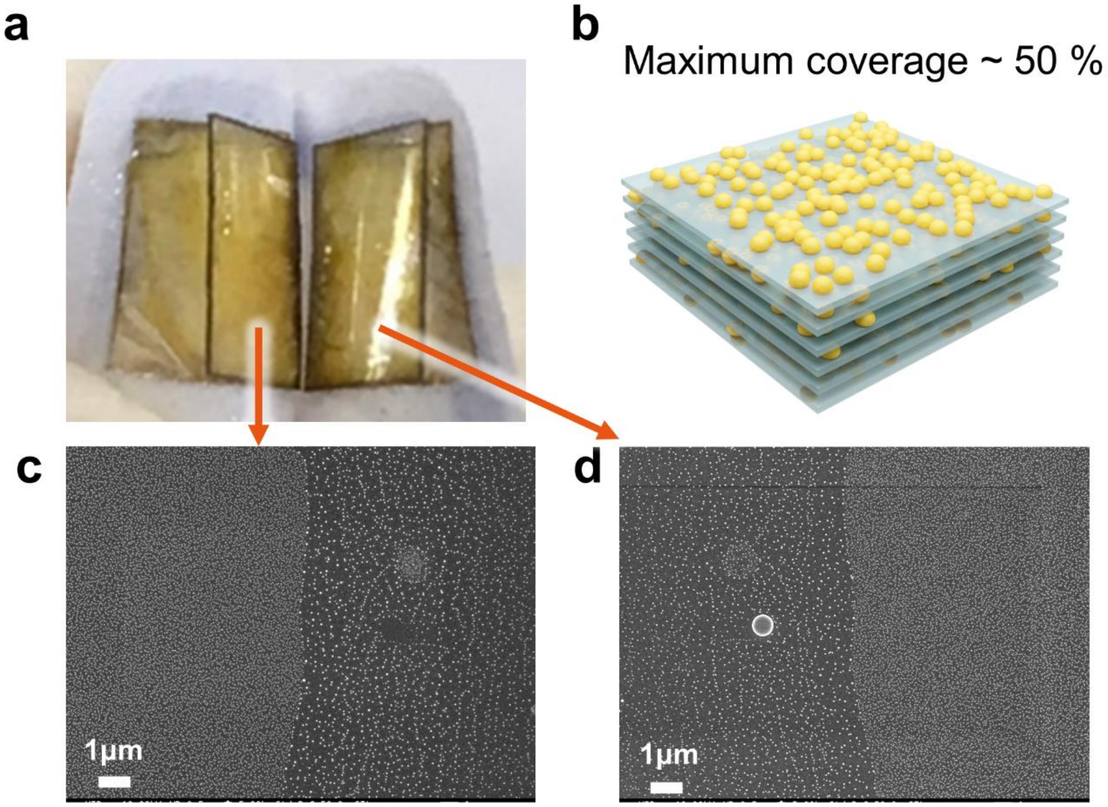


**Figure S2.** (a) Optical image of Ag NCs/mica sample (as-fabricated fresh samples). (b) Schematic of Ag NCs intercalated in mica layers. (c, d) Low-magnification SEM image of the left side (a) and right side of sample (a).

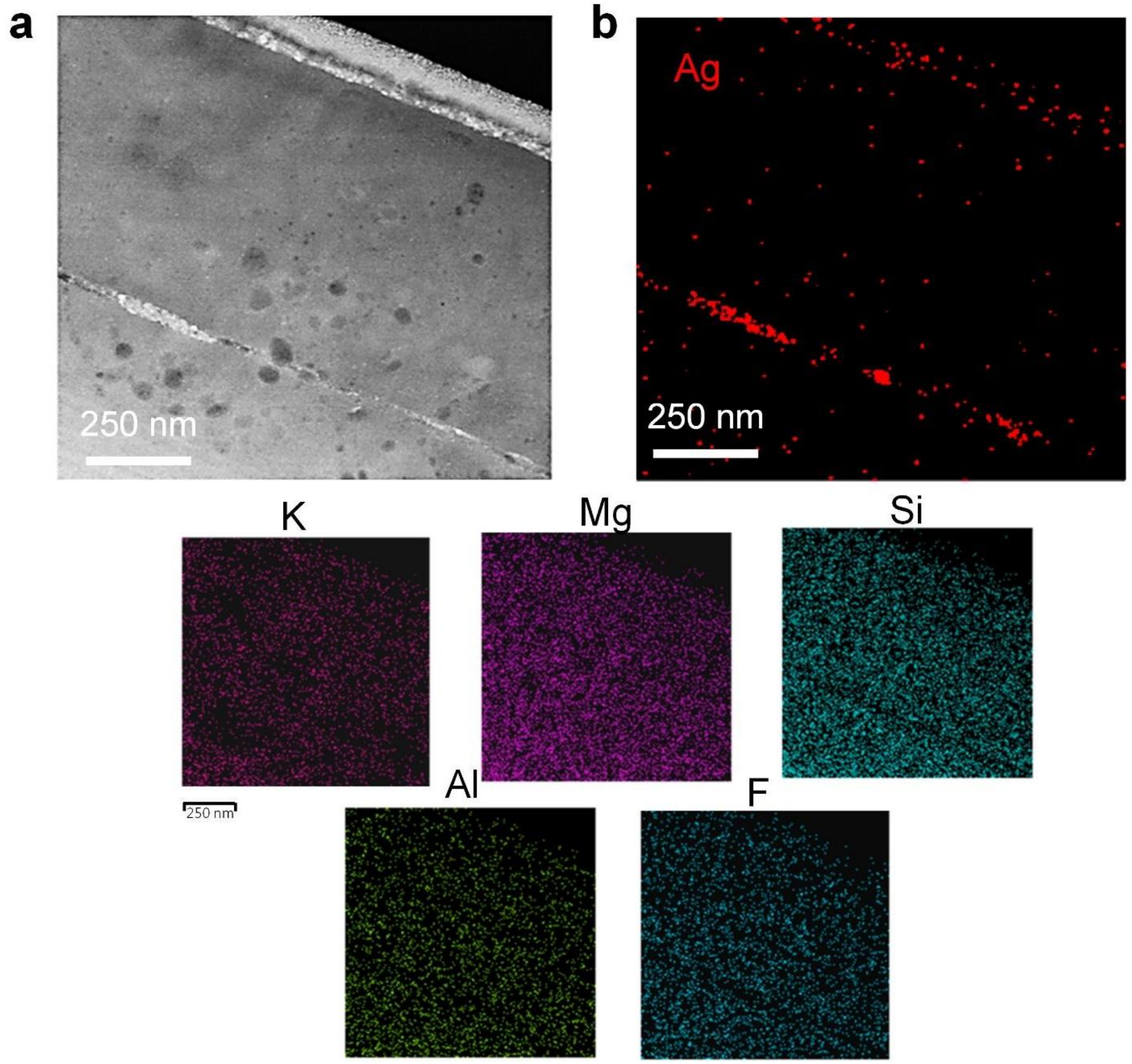


**Figure S3.** (a) Cross-section SEM represents the Ag NCs fabricated inside the mica crystal. (b) EDS-SEM of Ag element, showing randomly distributed Ag NCs in mica. The elements of mica, including K, Mg, Si, Al, and F were identified

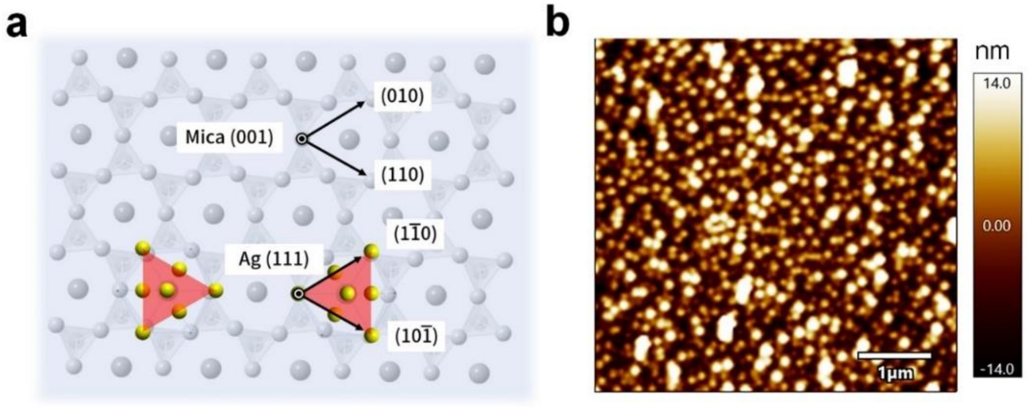


**Figure S4.** (a) The schematic diagram describes the relationship between Ag NCs grown on (00l) mica crystal. (b) 5×5 $\mu m^2$ AFM image taken on the Ag NCs/mica mesocrystal film.

To evaluate the optical property of epitaxial Ag NCs in mica, dielectric functions were measured in the wavelength range 300–1000 nm, as shown in **Figure S5**. The fitting results provide the real ($\varepsilon_1$) and imaginary ($\varepsilon_2$) parts of the wavelength-dependent dielectric function. The imaginary part of the Ag NCs' dielectric function can be comparable with Ag film/mica and Johnson & Christy data, corresponding to the optical loss of materials.[12,32-33] The large-scale epitaxial Ag NCs/mica mesocrystal has the benefits of large hot spots and dramatically enhances the Raman scattering signal.

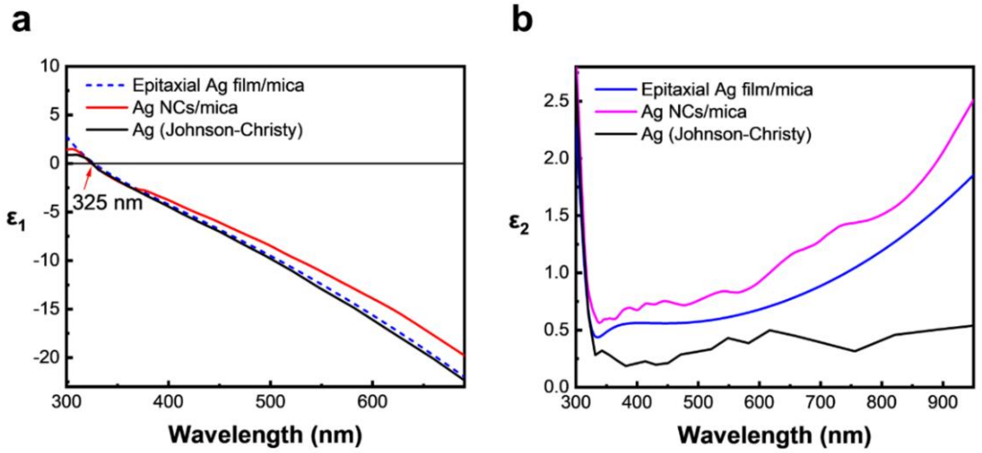


**Figure S5.** Optical properties of the epitaxial Ag NCs film compared with Johnson-Christy data and epitaxial Ag film. (a) and (b) Dielectric function ($\varepsilon_1$, $\varepsilon_2$) measured by spectroscopic ellipsometry (SE) for Ag films, Ag NCs/mica.

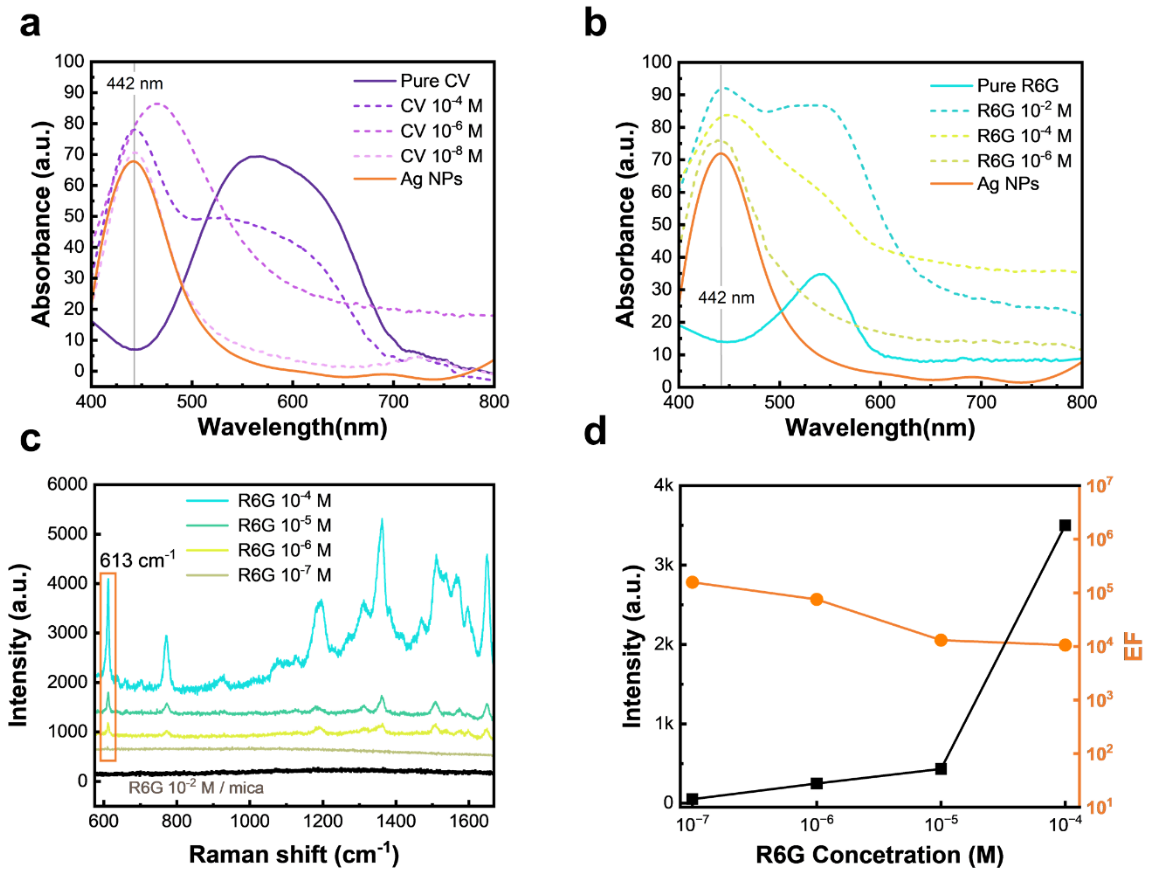


**Figure S6.** (a) Measured optical absorbance spectra of different CV and (b) R6G molecule concentrations supported on Ag NCs/mica display the LSPR resonance of 442 nm. (c) SERS spectra were obtained with different R6G concentrations. (d) The change of SERS intensity for various concentrations of R6G molecules at 613 $cm^{-1}$ Raman shifts (left-hand) and calculated enhancement factor (EF) of R6G on Ag NCs/mica SERS system (right-hand).

When Ag particles are closely spaced, interparticle coupling can induce hybridized plasmon modes, leading to dipole rotation or redistribution of the local field that deviating from the original incident polarization direction. To further investigate this hypothesis, we conducted control simulations with isolated Ag NCs, eliminating adjacent particles while keeping all other parameters constant (**Figure S7**). In these cases, the dipole orientation of the electric field remained aligned with the incident polarization, as expected for a single-particle dipolar response. This confirms that the deviations observed arise from particle–particle interactions rather than changes in material or excitation parameters.

To quantitatively assess the effect of mica thickness on near-field enhancement, we defined hotspots as regions where the normalized electric field intensity satisfies |E|/|E0|. Using this criterion, we analyzed the FDTD simulation data over a fixed 500 nm × 500 nm domain. For the 50 nm mica case, the hotspot density was approximately $1.04 \times 10^{12}$ hotspots/cm$^2$, whereas for the 700 nm case, it dropped to about $1.14 \times 10^{11}$ hotspots/cm². This represents more than a 9-fold increase in hotspot density when the mica thickness is reduced from 700 nm to 50 nm, due to stronger interlayer plasmonic coupling and reduced vertical dielectric separation.

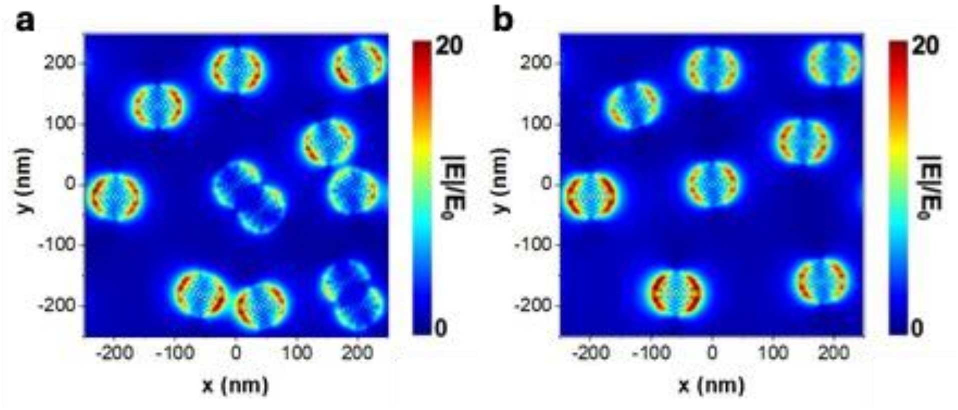


**Figure S7.** Simulated plane-view electric field distribution at the top Ag nanocrystals (NCs) with a mica thickness of 50 nm: (a) with adjacent Ag NCs; (b) without adjacent Ag NCs.

| SER platform | Reporter molecule | EF | LOD | Reference |
|---|---|---|---|---|
| Ag/Ag-doped $TiO_2$ | 4-mercaptobenzoic acid | $1.68 \times 10^6$ | $1.36\times10^7$molecules | [3] |
| Ag nanocubes | 1,4-benzenedithiol (1,4-BDT) | $1.34 \times 10^5$ | 0.05 M | [4] |
| Ag NPs | Crystal violet (CV) | $1\times10^5$ | $10^{-6}$ M | [5] |
| Ag-decorated $TiO_2$ nanorods | R6G | $3.1\times10^5$ | $10^{-7}$ M | [6] |
| Au coated on $TiO_2$ spheres | R6G | $1.4 \times 10^5$ | $10^{-6}$ M | [7] |
| Ag NCs/mica | Crystal violet | $8 \times 10^5$ | $10^{-7}$ M | This work |
| Ag NCs/mica | R6G | $1 \times 10^5$ | $10^{-6}$ M | This work |

**Table S1**: Comparison of enhancement factors.

## REFERENCE

(1) Lim, D. K.; Jeon, K. S.; Hwang, J. H.; Kim, H.; Kwon, S.; Suh, Y. D.; Nam, J. M. Highly Uniform and Reproducible Surface-Enhanced Raman Scattering from DNA-Tailorable Nanoparticles with 1-Nm Interior Gap. *Nat. Nanotechnol.***2011**, *6* (7), 452–460.

(2) Dubey, A.; Mishra, R.; Cheng, C. W.; Kuang, Y. P.; Gwo, S.; Yen, T. J. Demonstration of a Superior Deep-UV Surface-Enhanced Resonance Raman Scattering (SERRS) Substrate and Single-Base Mutation Detection in Oligonucleotides. *J. Am. Chem. Soc.***2021**, *143* (46), 19282–19286.

(3) Zhou, L.; Zhou, J; Lai, W.; Yang, X.; Meng, J; Su, L.; Gu, C.; Jiang, T.; Pun, E. W. B.; Shao, L.; Petti, L.; Sun, X. W.; Jia, Z.; Li, Q.; Han, J.; Mormile, P. Irreversible

accumulated SERS behavior of the molecule-linked silver and silver-doped titanium dioxide hybrid system. *Nat. commun.*2020, *11*(1), 1785.

(4) Zhou, S.; Li, J.; Gilroy, K. D.; Tao, J.; Zhu, C.; Yang, X.; Sun, X.; Xia, Y. Facile Synthesis of Silver Nanocubes with Sharp Corners and Edges in an Aqueous Solution. ACS Nano **2016**, *10*(11), 9861-9870.

(5) Doroshina, N. V.;Sttreletskiy, O. A.; Zavidovskiy, I. A.; Tatmyshevskiy, M. K.; Syuy, A. V.; Romanov. R.; Mishra, P.; Bobrovs, V.; Markeey, A. M.; Yakubovsky, D. I.; Veselova, I. A.; Arsenin, A. V.; Volkow, V. S.; Novikov, S. M. Crystallinity as a factor of SERS stability of silver nanoparticles formed by $Ar^+$ irradiation. *Heliyon***2024**, *10*(6),e27538.

(6) Fang, H., Zhang, C. X., Liu, L., Zhao, Y. M.; Xu, H. J. Recyclable three-dimensional Ag nanoparticle-decorated $TiO_2$ nanorod arrays for surface-enhanced Raman scattering. Biosens. Bioelectron. **2015**, *64*, 434-441.

(7) Li, X., Hu, H., Li, D., Shen, Z., Xiong, Q., Li, S. & Fan, H. J. Ordered array of gold semishells on TiO2 spheres: an ultrasensitive and recyclable SERS substrate. ACS Appl. Mater. Interfaces **2012**, *4*(4), 2180-2185.
